# Regional Fat Distributions Are Associated With Subclinical Right Ventricular Dysfunction in Adults With Uncomplicated Obesity

**DOI:** 10.3389/fcvm.2022.814505

**Published:** 2022-04-25

**Authors:** Jing Liu, Jing Li, Jianqun Yu, Chunchao Xia, Huaxia Pu, Wenzhang He, Xue Li, Xiaoyue Zhou, Nanwei Tong, Liqing Peng

**Affiliations:** ^1^Department of Radiology, West China Hospital, Sichuan University, Chengdu, China; ^2^Department of Endocrinology and Metabolism, West China Hospital, Sichuan University, Chengdu, China; ^3^MR Collaboration, Siemens Healthineers Ltd., Shanghai, China

**Keywords:** regional fat distributions, cardiovascular magnetic resonance, right ventricular, obesity, strain

## Abstract

**Objective:**

Obesity is a prominent public health problem that has increased cardiovascular mortality risks. However, the specific effects of obesity, independent of comorbidities, on cardiac structure and function have not been well clarified, especially those effects on the right ventricle (RV). Cardiovascular magnetic resonance (CMR) tissue tracking can assess detailed RV mechanical features. This study aimed to evaluate RV strain using CMR in uncomplicated obese adults and assess its association with fat distributions.

**Methods:**

A total of 49 obese patients and 30 healthy controls were included. The RV global systolic function and strain parameters based on CMR were assessed. Body fat distributions were measured with dual X-ray absorptiometry. RV function indices of obese patients were compared with those of healthy controls. Correlations among related body fat distribution parameters and RV function indices were conducted with multivariable linear regression.

**Results:**

Compared with healthy controls, the obese group had impaired RV strain with lower global longitudinal peak strain (PS), longitudinal peak systolic strain rate (PSSR), circumferential and longitudinal peak diastolic strain rates (PDSR) (all *P* < 0.05), while LV and RV ejection fractions were not significantly different between the two groups (*P* > 0.05). Multivariable linear regression analysis demonstrated that android fat% was independently associated with longitudinal PS (β = −0.468, model R^2^ = 0.219), longitudinal PDSR (β = −0.487, model R^2^ = 0.237), and circumferential PSSR (β = −0.293, model R^2^ = 0.086). Trunk fat% was independently associated with longitudinal PSSR (β = −0.457, model R^2^ = 0.209). In addition, the strongest correlations of circumferential PDSR were BMI and gynoid fat% (β = −0.278, β = 0.369, model R^2^ = 0.324).

**Conclusions:**

Extensive subclinical RV dysfunction is found in uncomplicated obese adults. BMI, as an index of overall obesity, is independently associated with subclinical RV dysfunction. In addition, central obesity (android fat and trunk fat distributions) has a negative effect on subclinical RV function, while peripheral obesity (gynoid fat distribution) may have a positive effect on it.

**Clinical Trials Registration:**

Effect of lifestyle intervention on metabolism of obese patients based on smart phone software (ChiCTR1900026476).

## Introduction

Overweight and obesity have rapidly increased global disease burden over the last decades. To date, no country has successfully reversed this obesity epidemic (1, 2). Since the beginning of the 21st century, obesity has become an epidemic in China caused by rapid economic growth with an estimated increase in prevalence of obesity of 0.32% per year (3). Obesity-related comorbidities, such as diabetes, hypertension, and coronary heart disease, are major risk factors that contribute to heart failure. However, in obese subjects with no clinically apparent cardiovascular risk factors, subclinical structural and functional changes can also be noted, which predisposes these individuals to heart failure at some point (4, 5). Previous studies examining cardiac function and the cardiovascular system have mainly focused on obese patients with comorbidities, while only few researches have focused on obese subjects without clinical signs or comorbidities. Specific right ventricular (RV) functional changes on CMR have not been studied in this subset of obese patients.

RV function assessment has been known to provide a prognostic role in several cardiac diseases (6). Previous echocardiographic studies evaluated RV functional strain in obese adults (4, 5, 7, 8). However, a review compared cardiac strain evaluations using cardiac magnetic resonance (CMR) and speckle tracking echocardiography (STE) and found that the STE technique had a major limitation, which greatly depended on image quality. Indeed, compared with CMR, echocardiographic images had lower signal-to-noise ratio (SNR) and inadequate imaging of some myocardial segments, especially in the distal part of the ultrasound sector (9). In obese subjects, signal interference due to excessive adiposity makes echocardiography more challenging. The advantages of CMR include larger fields of view, higher SNR, and 3-dimensional imaging of the heart, enabling the assessment of ventricular geometry and function with high accuracy and reproducibility (10). In clinical practice, ejection fraction (EF) is most widely used in evaluating global ventricular function; however, EF might not be a sensitive indicator of early RV dysfunction in obese subjects (7). Currently, the CMR tissue tracking technique is widely used as a subclinical myocardial dysfunction indicator for its high sensitivity when measuring global and regional cardiac mechanics through tracking myocardial motion (11). According to our literature search, only one article on children with uncomplicated obesity using CMR was identified. That article indicated that obese/overweight children had greater RV mass indices and lower RV free wall longitudinal strain (12). Until now, no studies have documented RV strain changes in obese adults with no clinical signs or comorbidities using CMR tissue tracking.

Body mass index (BMI) is the most widely used index of general obesity and is associated with other cardiovascular disease risks. Previous studies have found that different regions of fat deposition have various effects on the heart. For example, one study showed that visceral fat rather than subcutaneous fat was significantly associated with decreased RV strain (13). Dual X-ray absorptiometry (DXA) has also been extensively applied as it allows to accurately assess regional fat distributions, such as fat of the android, gynoid, trunk, upper and lower extremities, and visceral regions. A study examining the relationship between regional body fat and LV subendocardial viability ratios indicated that subjects with abdominal fat distribution (android-to-gynoid fat mass ratio) had poorer LV function (14). To our knowledge, no studies have focused on the association between regional fat distributions on RV function in obese individuals with no clinical signs or comorbidities. We aimed to evaluate RV functional changes seen on CMR in obese adults with no clinical signs or comorbidities and the association between RV strain and fat distributions.

## Methods and Materials

### Study Population

We prospectively recruited 49 obese subjects defined by a BMI ≥ 27.5 kg/m^2^ (ranging from 27.5 to 34.9 kg/m^2^) and 30 healthy volunteers (18.5 ≤ BMI ≤ 23.0 kg/m^2^) between 18 and 60 years old from September 2019 to September 2021. Subjects were excluded if they had any of the following conditions: hypertension or diabetes measured by oral glucose tolerance; history of cardiovascular diseases or history of any cardiovascular procedures; major systemic diseases that could affect the myocardium, such as connective tissue diseases and sarcoidosis; endocrine disease, such as hyperthyroidism and hypothyroidism; metabolic diseases, such as a history of alcohol abuse or amyloidosis; obstructive sleep apnea; any contraindication to CMR imaging. The study complied with the Declaration of Helsinki and was approved by the Institutional Review Board of the West China Hospital in Sichuan University. Written informed consent was obtained from all study participants.

### Baseline Data Collection

Baseline data of the participants were collected, including medical history, anthropometric measurements (weight and height), heart rate, and blood pressure. Fasting blood glucose and serum lipid profiles, including triglycerides, total cholesterol, high-density lipoprotein (HDL), and low-density lipoprotein (LDL), were also measured.

### Assessment of Obesity

Waist circumference (WC) was measured at the midway between the last rib and the iliac crest, and hip circumference (HC) was measured at the largest diameter of the hip. WC and HC were measured to the nearest 0.5 cm. The waist-to-hip ratio and waist-to-height ratio were calculated. Body mass index (BMI) (kg/m^2^) was calculated as weight (kg) divided by height squared (m^2^). According to the Chinese criteria, BMI was categorized into the following three groups: healthy weight (18.5-23.0 kg/m^2^), overweight (23.0-27.5kg/m^2^), and obese (≥27.5kg/m^2^) (15). Total fat, android fat, gynoid fat, trunk fat, upper and lower extremities fat, and visceral fat mass (g) were measured using DXA (Lunar iDXA, GE Medical Systems Lunar, Madison, USA). Specifically, the region of interest (ROI) of android fat distribution was defined from the pelvic cut (lower boundary) to above the pelvis cut by 20% of the distance between the iliac crest and chin (upper boundary). The gynoid fat distribution ROI upper boundary was 1.5 times the height of the android ROI below the iliac crest to a line equal to twice the height of the android fat distribution ROI (lower boundary) (16). Percentage of fat mass in android, gynoid, trunk, peripheral, upper extremities and lower extremities, and visceral regions reflect fat deposition in the corresponding regions, relative to total fat mass. Peripheral fat mass was calculated as the sum of upper and lower extremities fat mass. Percentage of fat mass in trunk, android, and visceral regions (also expressed as trunk fat%, android fat%, and visceral fat%) were indices predictive of central obesity. While Percentage of fat mass in gynoid, peripheral, upper extremities and lower extremities regions (also expressed as gynoid fat%, peripheral fat%, upper extremities fat%, and lower extremities fat%) were indices predictive of peripheral obesity.

### CMR Protocol

CMR examinations were performed using a 3 Tesla whole-body scanner (MAGNETOM Skyra, Siemens Healthcare, Erlangen, Germany) with an 18-channel phased-array body coil on patients in a supine position. With a standard ECG-triggering device, data was acquired during the end-expiratory breath-hold period. A segmented breath-hold balanced steady-state free precession (bSSFP) sequence was used to obtain 8–14 contiguous cine images from the base to the apex in the short-axis view and the two- and four-chamber cine images in the long-axis view. The bSSFP parameters are as follows TR/TE = 3.3/1.22 ms, flip angle = 41°, slice thickness = 8 mm, field of view = 360 × 320 mm^2^, matrix size = 208 × 166, and a temporal resolution = 39.34 ms.

### CMR Image Analysis

All CMR data was imported to commercially available software (CVI 42 version 5.11.3, Circle Cardiovascular Imaging Inc., Calgary, Canada). Two radiologists with more than 3 years of CMR experience analyzed the measurements and were blinded to the subject status (obesity vs. control).

#### Epicardial Adipose Tissue Quantification

Epicardial adipose tissue (EAT) represents high-signal intensity region between the myoepicardium and parietal pericardium. The EAT volume was measured on the short-axis cine slices during the end-diastolic phase. The myoepicardial and parietal pericardial contours were manually delineated per slice, extending from the mitral valve hinge down to the ventricular apex. High-signal intensity regions between the myoepicardium and parietal pericardium were semi-automatically traced and calculated, excluding blood vessels.

#### Global Ventricular Geometry and Function

The endocardial and epicardial contours of the right ventricle (RV) and left ventricle (LV) myocardium on the short-axis cine images were manually traced during the end-diastolic and end-systolic phases of the CVI42 short-3D module software. The global conventional functional parameters, namely EF, end-diastolic volume (EDV), end-systolic volume (ESV), and LV mass at end-diastole, were automatically computed. In addition, the average LV regional values for 16 myocardial segment thicknesses (American Heart Association standard segmentation model) were also automatically computed. Finally, the LV thickness and interventricular septal (IVS) thickness averages were calculated.

#### RV and LV Strain

**Figure 1:**
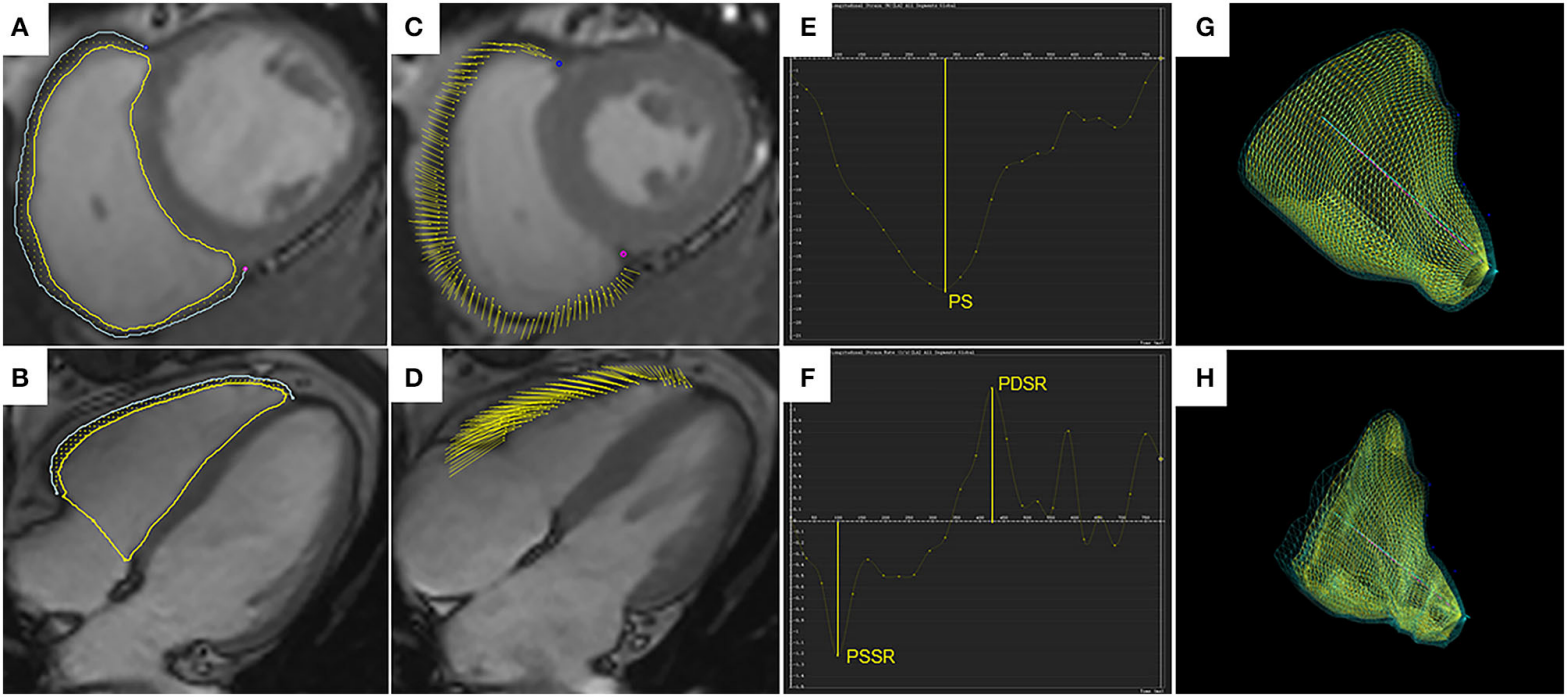
Right ventricular tissue tracking using cardiac magnetic resonance imaging. Right ventricular (RV) contours are delineated on the short-axis SSFP image **(A)** and the four-chamber image **(B)**, shown for the end-diastolic phase. Yellow and blue lines represent RV endocardial and epicardial borders, and yellow dots between the borders represent myocardial points. **(C,D)** demonstrate myocardial points motion from the end-diastolic phase to the end-systolic phase using tissue tracking. **(E,F)** show strain curve and strain rate curve. The peak strain (PS) and strain rates (PSSR and PDSR) can be acquired in the curves. 3D models of the RV in the end-diastolic phase **(G)** and the end-systolic phase **(H)**. SSFP, steady-state free precession; PS, peak strain; PSSR, peak systolic strain rate; PDSR, peak diastolic strain rate; 3D, three-dimensional.

The long-axis 4-chamber, 2-chamber and short-axis cine slices were transferred to the three-dimensional tissue tracking module for RV and LV myocardial strain analysis. The endocardial and epicardial contours were manually delineated per slice during the end-diastolic phase in all series, and the papillary muscles and moderator bands were excluded (Figure 1). Strain is defined as the degree of myocardial deformation from its initial length (*L*_0_, in end-diastole) to its maximum length (*L*, in end-systole): *myocardial strain* = (*L* − *L*_0_)/*L*_0_. Strain rate represents the rate of length shortening (17). For different directions of myocardial deformation, the RV and LV global myocardial strain parameters, including the radial, circumferential, and longitudinal peak strains (PS), peak systolic strain rates (PSSR), and peak diastolic strain rates (PDSR) can be calculated. Since RV radial deformation values had low reproducibility and high variability due to the complex morphologic structure and relatively thin ventricular wall, the related parameters (radial PS, PSSR, and PDSR) were excluded.

#### Reproducibility

Intra- and inter-observer variabilities for the EAT and RV myocardial strain indices were analyzed in 20 random subjects, including 12 obese patients and 8 healthy controls. To determine intra-observer variability, one radiologist measured the same image within a 1-month interval. To evaluate the inter-observer variability, the second radiologist, who was blinded to the first observer's results, re-analyzed the measurements.

### Statistical Analysis

All statistical analyses were performed using SPSS software (version 23, IBM, Armonk, Armonk, New York, USA). Continuous data with normal distributions were compared between the obese and healthy groups with the student's *t*-test and were expressed as the mean ± SD. Binary variables were analyzed using the cross tabs Chi-square test. Correlations between RV functional parameters and LVEF and LV geometry (mass and thickness) and IVS thickness were assessed in obese patients. Pearson's correlation coefficient was used to determine the correlations between regional fat, blood lipids, and RV functional parameters (global function and strain indices) in whole population. Stepwise multivariable linear regression was used to estimate the associations between regional fat distributions and RV functional parameters in whole population. Related fat distribution, including android fat%, gynoid fat%, trunk fat%, peripheral fat%, visceral fat%, EAT were entered in univariable analyses. Variables with statistical significance in the univariable analyses were then included in a stepwise multivariable analysis. In order to determine the association between obesity and RV function independent of growth differences, age and sex were added in multivariable analyses. The intraclass correlation coefficient (ICC) was used to evaluate both inter-and intra-observer variabilities. A *P* < 0.05 indicated statistical significance.

## Results

### Baseline Characteristics

This study included 49 obese subjects (BMI 29.9 ± 2.0 kg/m^2^) and 30 healthy controls (19.7 ± 1.1 kg/m^2^), and the baseline characteristics of the patients are shown in Table 1. Mean age, height, male proportion, and heart rate were compared and the differences between the two groups were not statistically significant. Compared with healthy controls, obese subjects had higher blood pressure, although it was within the normal range. For blood parameters, the obese group had higher fasting blood glucose, triglycerides, total cholesterol, and LDL, and lower HDL compared with the healthy group. The obese group had greater conventional fat indexes, including WC, HC, waist-to-hip ratio, and waist-to-height ratio compared to healthy group. Additionally, the obese group had greater DXA-related central fat deposition indexes, including trunk fat%, visceral fat%, and android fat% compare those of healthy group. In contrast, they had lower DXA-related peripheral fat deposition indexes including gynoid fat%, peripheral fat%, and lower extremities fat% compared with healthy individuals. The obese group had greater EAT than those of healthy group.

**Table 1:** Baseline characteristics of the study cohort.

**Variables**	**Controls** **(*n* = 30)**	**Obese patients** **(*n* = 49)**	***P*-value**
Demographics			
Male, *n* (%)	10 (33.3)	27 (55.1)	0.06
Age (years)	28.8 ± 7.1	32.6 ± 8.8	0.05
Height (cm)	164.4 ± 8.1	167.6 ± 9.4	0.130
Weight (kg)	53.2 ± 5.3	84 ± 11	<0.001^*^
BMI (kg/m^2^)	19.7 ± 1.1	29.9 ± 2.0	<0.001^*^
Hemodynamic variables			
Heart rate (bpm)	73.4 ± 8.1	73.7 ± 9.6	0.860
SBP (mmHg)	104 ± 12	123.5 ± 9.9	<0.001^*^
DBP (mmHg)	70.4 ± 8.1	78.9 ± 6.5	<0.001^*^
Laboratory data			
Fasting blood glucose (mmol/L)	4.7 ± 0.3	5.4 ± 0.7	<0.001^*^
Plasma triglycerides (mmol/L)	0.6 ± 0.2	1.9 ± 1.4	<0.001^*^
Total cholesterol (mmol/L)	3.9 ± 0.7	4.9 ± 1.1	<0.001^*^
HDL (mmol/L)	1.6 ± 0.3	1.3 ± 0.3	<0.001^*^
LDL (mmol/L)	2.1 ± 0.6	2.7 ± 0.8	<0.001^*^
Fat distribution			
EAT (cm^3^)	20.6 ± 7.9	48 ± 14	<0.001^*^
Total fat (kg)	12.0 ± 3.0	29.3 ± 5.7	<0.001^*^
Android fat%	5.7 ± 1.1	9.8 ± 1.5	<0.001^*^
Gynoid fat%	18.4 ± 2.9	14.9 ± 2.0	<0.001^*^
Trunk fat%	44.9 ± 4.6	57.7 ± 4.7	<0.001^*^
Peripheral fat%	47.5 ± 5.1	38.5 ± 5.0	
Upper extremities fat%	11.1 ± 0.2	10.5 ± 0.2	0.08
Lower extremities fat%	36.4 ± 4.9	28.0 ± 4.2	<0.001^*^
Visceral fat%	2.0 ± 1.4	4.9 ± 2.5	<0.001^*^
Waist circumference (cm)	72.1 ± 4.7	100 ± 11	<0.001^*^
Hip circumference (cm)	92.1 ± 3.9	107.3 ± 4.1	<0.001^*^
Waist-to-height ratio	0.78 ± 0.05	0.93 ± 0.09	<0.001^*^
Waist-to-hip ratio	0.4 ± 0.2	0.60 ± 0.06	<0.001^*^

*BMI, body mass index; SBP, systolic blood pressure; DBP, diastolic blood pressure; HDL, high-density lipoprotein; LDL, low-density lipoprotein; EAT, epicardial adipose tissue*.

* *P < 0.05*.

### Comparison of CMR Findings Between the Obese Subjects and Healthy Controls

**Figure 2:**
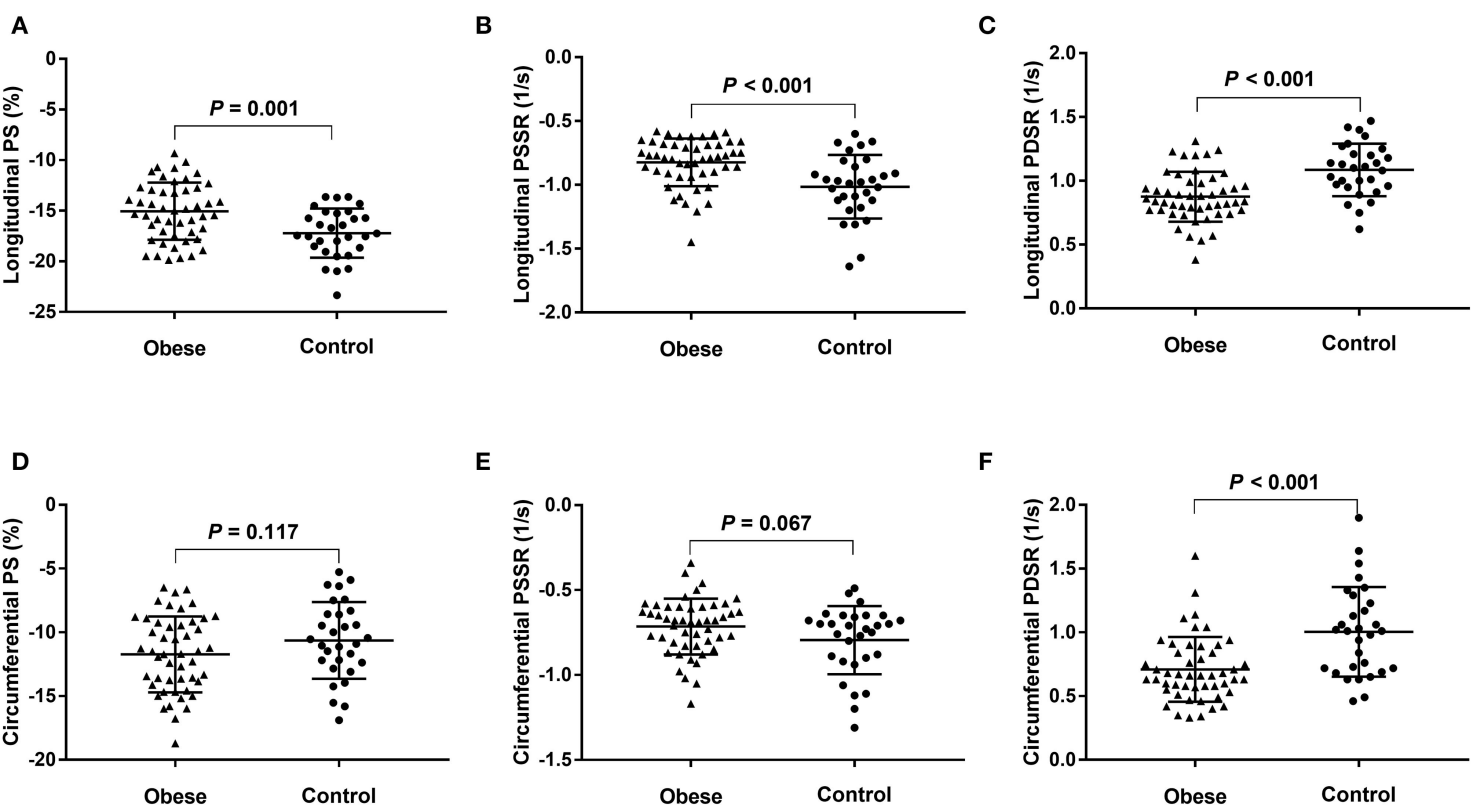
Dot plots comparing the right ventricular strain parameters of patients with obesity and normal controls. **(A)** Longitudinal PS, **(B)** longitudinal PSSR, **(C)** longitudinal PDSR, **(D)** circumferential PS, **(E)** circumferential PSSR, and **(F)** circumferential PDSR. PS, peak strain; PSSR, peak systolic strain rate; PDSR, peak diastolic strain rate.

The RVEF and LVEF were within the normal range (RVEF > 40% and LVEF > 50%) for all obese patients and neither were statistically different between the two groups (51.2 ± 4.3% vs. 50.9 ± 4.6%, *P* = 0.087; 60.9 ± 4.5% vs. 62.7 ± 4.6 %, *P* = 0.091, respectively) compared with the controls. Compared with the healthy controls, obese patients exhibited greater RV sizes (RVEDV, RVESV), LV sizes (LVEDV, LVESV), LV geometric parameters (LV mass and LV average thickness), and IVS average thickness. For RV strain, the obese group showed lower global longitudinal PS, PSSR and PDSR, circumferential PDSR, and preserved circumferential PS and PSSR compared with the control group (Figure 2). For LV strain, the obese group showed lower global longitudinal and circumferential PS and preserved radial PS compared with the control group (Table 2).

**Table 2:** Comparison of cardiac magnetic resonance parameters between two groups.

**Variables**	**Controls** **(*n* = 30)**	**Obese** **patients** **(*n* = 49)**	***P*-value**
RV global function			
RVEF	51.2 ± 4.3	50.9 ± 4.6	0.087
RVEDV (ml)	136 ± 30	163 ± 38	<0.001^*^
RVESV (ml)	67 ± 18	81 ± 24	<0.001^*^
RV strain			
Circumferential PS (%)	−10.6 ± 3.0	−11.7 ± 3.0	0.117
Circumferential PSSR (1/s)	−0.8 ± 0.2	−0.7 ± 0.2	0.067
Circumferential PDSR (1/s)	1.0 ± 0.3	0.7 ± 0.3	<0.001^*^
Longitudinal PS (%)	−17.2 ± 2.4	−15.1 ± 2.8	0.001^*^
Longitudinal PSSR (1/s)	−1 ± 0.4	−0.8 ± 0.2	<0.001^*^
Longitudinal PDSR (1/s)	1.1 ± 0.2	0.9 ± 0.2	<0.001^*^
LV global function			
LVEF	60.9 ± 4.5	62.7 ± 4.6	0.091
LVEDV (ml)	120 ± 24	158 ± 28	<0.001^*^
LVESV (ml)	49.1 ± 9.6	60 ± 14	<0.001^*^
LV mass (g)	73 ± 16	91 ± 20	<0.001^*^
LV average thickness (mm)	5.6 ± 0.7	6.0 ± 0.8	0.016^*^
IVS average thickness (mm)	5.7 ± 0.4	6.7 ± 0.9	<0.001^*^
LV strain			
Longitudinal PS (%)	−15.8 ± 1.9	−13.5 ± 2.9	<0.001^*^
Circumferential PS (%)	−20.7 ± 1.9	−19.6 ± 2.0	0.018^*^
Radial PS (%)	34.6 ± 5.5	32.1 ± 5.5	0.057

*RV, right ventricular; EF, ejection fraction; EDV, end diastolic volume; ESV, end systolic volume; PS, peak strain; PSSR, peak systolic strain rate; PDSR, peak diastolic strain rate; LV, left ventricular; IVS, interventricular septum*.

* *P < 0.05*.

### Associations Between RV Strain Parameters, Biventricular Geometries, and Global Systolic Function in Obese Individuals

Among obese subjects, the RVEF had a positive correlation with the circumferential PS and longitudinal PS (r = 0.419 and r = 0.328); whereas RV size (RVEDV and RVESV), LV size (LVEDV and LVESV), LV mass, LV average thickness, and ISV average thickness showed weak-moderate negative correlations with RV strain indices (r = −0.3 to −0.5). Among them, LV average thickness was strongest for the longitudinal PS (r = −0.504). In addition, LVEF was positively correlated with RV longitudinal PS, PSSR, and PDSR (r = 0.3 to 0.4); LV longitudinal, circumferential, and radial PS had positive correlations with the RV strain parameters (r = 0.3 to 0.4) (Table 3).

**Table 3:** Association (correlation coefficients) between cardiac geometry, function, and RV strains in obese patients.

	**RV Circumferential**			**RV Longitudinal**		
	**PS**	**PSSR**	**PDSR**	**PS**	**PSSR**	**PDSR**
RVEF	0.419^**^	0.252	0.154	0.328^*^	0.136	0.249
RVEDV	−0.439^**^	−0.395^**^	−0.398^**^	−0.365^*^	−0.178	−0.319^*^
RVESV	−0.472^**^	−0.401^**^	−0.362^*^	−0.381^**^	−0.190	−0.339^*^
LVEF	0.130	0.112	0.007	0.311^*^	0.323^*^	0.366^**^
LVEDV	−0.315^*^	−0.232	−0.313^*^	−0.213	−0.295^*^	−0.248
LVESV	−0.319^*^	−0.281	−0.251	−0.351^*^	−0.397^**^	−0.417^**^
LV mass	−0.292^*^	−0.223	−0.333^*^	−0.412^**^	−0.311^*^	−0.185
LV thickness	−0.362^*^	−0.228	−0.369^**^	−0.504^**^	−0.266	−0.198
IVS thickness	−0.295^*^	−0.147	−0.289^*^	−0.444^**^	−0.201	−0.192
LV longitudinal PS	0.303^*^	0.326^*^	0.286^*^	0.186	0.270	0.257
LV circumferential PS	0.301^*^	0.357^*^	0.154	0.217	0.271	0.310^*^
LV radial PS	0.438^**^	0.440^**^	0.224	0.375^**^	0.423^**^	0.348^*^

*Longitudinal/circumferential PS and PSSR are calculated as absolute values. RV, right ventricular; EF, ejection fraction; EDV, end diastolic volume; ESV, end systolic volume; LV, left ventricular; IVS, interventricular septum; PS, peak strain; PSSR, peak systolic strain rate; PDSR, peak diastolic strain rate*.

* *P < 0.05 and*

** *P < 0.01*.

### Association Between Regional Fat Distributions and Cardiovascular Risk Factors in Whole Population

BMI, EAT, android fat%, and trunk fat% were positively correlated with fasting blood glucose, triglycerides, total cholesterol, and LDL (r = 0.3 to 0.6), while were negatively correlated with HDL (r = −0.4 to −0.6). In contrast, gynoid fat% had negative associations with triglycerides, total cholesterol, and LDL (−0.2 to −0.5), while had a positive association with HDL (r = 0.484); peripheral fat% was negatively associated with fasting blood glucose and total cholesterol (r = −0.236; r = −0.232) (Table 4).

**Table 4:** Association between regional fat distributions and cardiovascular risk factors in whole population.

	**Fasting blood glucose**	**Plasma triglycerides**	**Total cholesterol**	**HDL**	**LDL**
BMI	0.445^**^	0.548^**^	0.403^**^	−0.509^**^	0.296^**^
EAT	0.446^**^	0.457^**^	0.359^**^	−0.387^**^	0.335^**^
Android fat%	0.415^**^	0.637^**^	0.429^**^	−0.549^**^	0.336^**^
Gynoid fat%	−0.209	−0.530^**^	−0.305^**^	0.484^**^	−0.227^*^
Trunk fat%	0.354^**^	0.644^**^	0.444^**^	−0.554^**^	0.334^**^
Peripheral fat%	−0.236^*^	−0.204	−0.232^*^	0.076	−0.178
Visceral fat%	0.202	0.531^**^	0.372^**^	−0.474^**^	0.301^**^

*LDL, low-density lipoprotein; HDL, high-density lipoprotein; BMI, body mass index; EAT, epicardial adipose tissue*.

* *P < 0.05 and*

* *P < 0.01*.

### Association Between Adiposity and RV Functional Parameter in Whole Population

**Figure 3:**
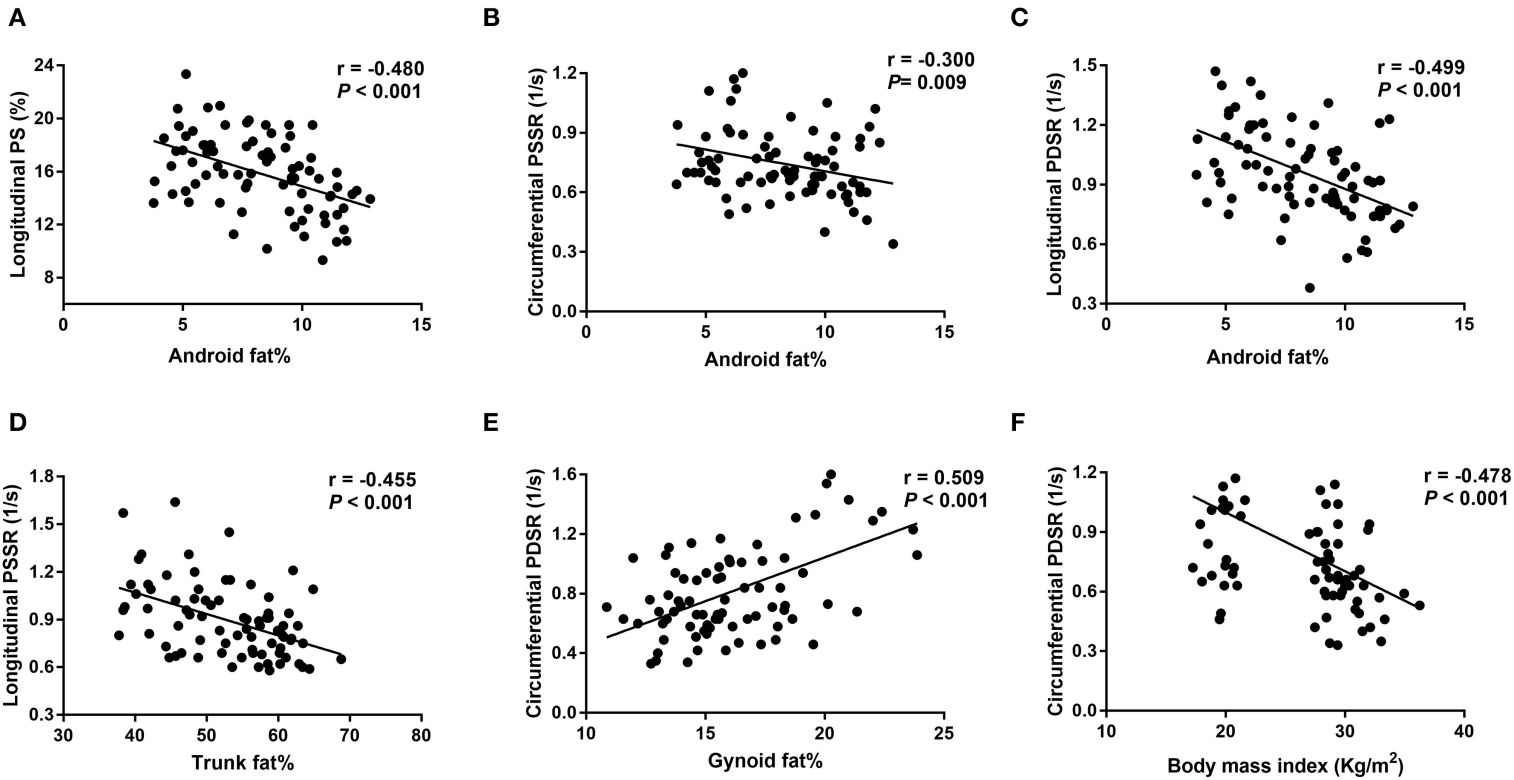
Correlations between fat distributions and right ventricular strain parameters. **(A-C)** Show negative correlations between android fat% and longitudinal PS, longitudinal PDSR and circumferential PSSR; **(D)** shows a negative correlation between trunk fat% and longitudinal PSSR; **(E)** shows a positive correlation between gynoid fat% and circumferential PDSR; **(F)** shows a negative correlation between body mass index and circumferential PDSR. PS, peak strain; PSSR, peak systolic strain rate; PDSR, peak diastolic strain rate. Longitudinal PS and longitudinal/circumferential PSSR are showed as absolute values.

Univariable analysis showed that BMI, android fat% and trunk fat% were negatively associated with longitudinal PS, longitudinal and circumferential strain rate (PSSR and PDSR) (r = −0.3 to −0.5). Visceral fat% and EAT were negatively associated with circumferential PDSR and longitudinal PS, PSSR and PDSR (r = −0.3 to −0.4). In contrast, gynoid fat% and peripheral fat% were positively associated with circumferential PDSR, and longitudinal PS, PSSR and PDSR (r = 0.4 to 0.5); gynoid fat% was positively associated with RVEF (r = 0.264) (Table 5 and Figure 3).

**Table 5:** Association between regional fat distributions and RVEF and strain parameters in whole population.

	**RVEF**	**RV Circumferential**			**RV Longitudinal**		
		**PS**	**PSSR**	**PDSR**	**PS**	**PSSR**	**PDSR**
BMI	−0.045	0.118	−0.287^*^	−0.478^**^	−0.380^**^	−0.428^**^	−0.464^**^
EAT	0.040	0.235	−0.088	−0.335^**^	−0.370^**^	−0.327^**^	−0.442^**^
Android fat%	−0.090	0.041	−0.300^**^	−0.485^**^	−0.480^**^	−0.476^**^	−0.499^**^
Gynoid fat%	0.264^*^	0.060	0.174	0.509^**^	0.394^**^	0.367^**^	0.391^**^
Trunk fat%	−0.130	0.037	−0.279^*^	−0.450^**^	−0.455^**^	−0.455^**^	−0.462^**^
Peripheral fat%	0.216	0.016	0.216	0.431^**^	0.451^**^	0.440^**^	0.446^**^
Visceral fat%	−0.139	0.083	−0.044	−0.271^*^	−0.362^**^	−0.340^**^	−0.372^**^

*Longitudinal/circumferential PS and PSSR are calculated as absolute values. RV, right ventricular; EF, ejection fraction; PS, peak strain; PSSR, peak systolic strain rate; PDSR, peak diastolic strain rate; BMI, body mass index; EAT, epicardial adipose tissue*.

* *P < 0.05 and*

** *P < 0.01*.

Multivariable linear regression analysis demonstrated that android fat% was independently associated with longitudinal PS (β = −0.468, *P* < 0.001, model R^2^ = 0.219), longitudinal PDSR (β = −0.487, *P* < 0.001, model R^2^ = 0.237), and circumferential PSSR (β = −0.293, *P* = 0.009, model R^2^ = 0.086). Trunk fat% was independently associated with longitudinal PSSR (β = −0.457; *P* < 0.001, model R^2^ = 0.209). In addition, the strongest correlates of circumferential PDSR were BMI and gynoid fat% (β = −0.278, *P* = 0.016; β = 0.369, *P* = 0.002; model R^2^ = 0.324) (Table 6).

**Table 6:** Multivariable linear regression analysis of association between regional fat distributions and RV strains in whole population.

**Independent** **variables**	**Factors in models**	**R square**	**Adjusted R square**	**B**	**β**	***P*-value**
Longitudinal PS	Android fat%	0.219	0.209	−0.549	−0.468	<0.001
Longitudinal PSSR	Trunk fat%	0.209	0.199	−0.013	−0.457	<0.001
Longitudinal PDSR	Android fat%	0.237	0.227	−0.048	−0.487	<0.001
Circumferential PSSR	Android fat%	0.086	0.074	−0.022	−0.293	0.009
Circumferential PDSR	BMI	0.324	0.306	−0.017	−0.278	0.016
	Gynoid fat%			0.042	0.369	0.002

*Longitudinal PS and longitudinal/circumferential PSSR are showed as absolute values. RV, right ventricular; PS, peak strain; PSSR, peak systolic strain rate; PDSR, peak diastolic strain rate; BMI, body mass index*.

### Intra-observer and Inter-observer Variability

The intra- and inter-observer variability for EAT quantifications and strain parameters are summarized in Table 7. The ICCs for intra- and inter-observer variability were 0.833 and 0.790 for EAT, respectively. There were good intra- and inter-observer agreements for the RV strain parameters (ICC = 0.794-0.921 and 0.773-0.920, respectively), and circumferential and longitudinal strains had better repeatability than the strain rates.

**Table 7:** Comparison of inter- and intra-observer variability among epicardial adipose tissue and RV peak strain parameters.

	**Intra-observer (*****n*** **=** **20)**		**Inter-observer (*****n*** **=** **20)**	
	**ICC**	**95% CI**	**ICC**	**95% CI**
EAT	0.883	0.705-0.957	0.790	0.577-0.923
Circumferential PS	0.921	0.864-0.955	0.920	0.862-0.976
Longitudinal PS	0.906	0.839-0.946	0.891	0.808-0.939
Circumferential PSSR	0.840	0.609-0.941	0.782	0.490-0.917
Longitudinal PSSR	0.794	0.513-0.922	0.773	0.468-0.914
Circumferential PDSR	0.851	0.627-0.945	0.819	0.555-0.933
Longitudinal PDSR	0.889	0.711-0.960	0.774	0.474-0.914

*EAT, epicardial adipose tissue; PS, peak strain; PSSR, peak systolic strain rate; PDSR, peak diastolic strain rate; ICC, intraclass correlation coefficient; CI, confidence interval*.

*All P < 0.001*.

## Discussion

In this study, we compared RV functional parameters in adults with uncomplicated obesity with those in normal controls using CMR and assessed associations between fat distributions and RV function in whole population. The main findings were as follows: (1) although the RVEF was within the normal range in both groups, the subjects with obesity had impaired RV myocardial contractility manifestied by lower longitudinal PS, longitudinal PSSR, and circumferential and longitudinal PDSR; (2) Decreased LV longitudinal and circumferential PS and preserved LVEF in obese group compared with healthy group. (3) the individuals with obesity had cardiac remodeling with greater RV size, LV size, LV myocardial mass, and LV wall thickness compared with controls; (4) the impaired subclinical RV function was associated with cardiac remodeling; (5) BMI, as an index of overall obesity, was independently associated with subclinical RV dysfunction. In addition, central fat distribution indicators (android fat% and trunk fat%) were negatively correlated with subclinical RV function, whereas peripheral fat distribution indicator (gynoid fat%) was positively correlated with it. Until now, no studies have documented RV strain changes in obese adults with no clinical signs or comorbidities using CMR tissue tracking and the associations between DXA-related fat distributions and RV function.

### Obesity and RV Dysfunction

Our study revealed no statistical differences for RVEFs between the two groups, indicating a lack of global right ventricular systolic functional impairment. This result is consistent with that of previous echocardiographic studies of obese adults without known cardiovascular diseases (4, 7). Nevertheless, a CMR study exhibited lower RVEF in obese group compared with the control group, in which partial anticipants were diagnosed with hypertension or diabetes (18). The difference between two CMR studies may be attributed to the multi-ethnic participants that were older in age (ranging from 45 to 84 years old) and had obesity-related complications in previous study. Furthermore, our study showed decreased RV longitudinal PS and strain rates (PSSR and PDSR) of obese participants compared with those of normal controls. These results are similar with those of several other echocardiographic studies in obese adults (4, 5, 7, 8). There are only subendocardial and subepicardial layers in the RV myocardium. Longitudinal deformation is primarily caused by the shortening of longitudinal myocardial fibers located in the subendocardial layer (17). Obesity can alter myocardial perfusion, resulting in myocardial ischemia, and meanwhile the subendocardial layer is vulnerable to microvascular ischemia (19). Moreover, our study also revealed impaired circumferential RV mechanics, represented by decreased circumferential PDSR in obese group compared with those in healthy group, which has not been previously reported. Notably, the differences in circumferential PS and PSSR between two groups were not statistically significant, confirming that PDSR is more sensitive to subclinical myocardial dysfunction than either PS or PSSR in obesity. Circumferential strain largely reflects the circumferentially oriented myofibers in the subepicardial layer (20) which might indicate that the myocardial injury is not only in the subendocardial layer but also in the subepicardial layer of the RV myocardium in obese patients. In summary, subclinical RV functional impairment occurred before decrease in RVEF in obese participants.

In addition, obese participants had greater RV volumes (RVESV and RVEDV) compared with those of the normal subjects in our study. A echocardiographic study demonstrated that RV volumes significantly increased only in the severely obese group (BMI = 46.8 ± 11 kg/cm^2^), but not in mild-to-moderate obesity (7). Another echocardiographic study showed no changes in RV volumes in mild obese group (4). The results indicate that CMR is more sensitive than echocardiography to measure RV volume. In fact, echocardiography with limited acoustic window, low spatial resolutions, and compromised geometric assumptions, is inaccurate for RV geometric parameter evaluations (21). Meanwhile, RV volumes were negatively associated with RV strain and strain rates in our study. RV dilation is caused by increased total blood volume and cardiac output due to the high metabolic activity of obesity (22). RV dilation can result in contractility impairments due to increased ventricular wall stress (23).

Our study might find ventricular-ventricular interactions in obese individuals. LV myocardial remodeling (greater chamber size, wall thickness, and mass occurred) in obese adults. LV myocardial remodeling was negatively associated with the RV strain parameters, which indicated that RV function could be impacted by LV myocardial remodeling through ventricular-ventricular interactions. Similar results were also reported in a CMR study with uncomplicated obese children (12). In an experimental study of dogs, the RV free wall was electrically isolated and showed that despite stopping the RV free wall pacing in diastole, more than half of the RV systolic pressure and pulmonary flow were obtained in the subsequent heart beat (24). Another study demonstrated that LV shortening contributed to RV stroke exertion when the RV free wall was replaced with a non-contractile patch (25). In other words, LV contraction directly impacts RV function. In our study, LV global longitudinal and circumferential PS decreased in obese group compared with healthy group, indicating LV contraction impairment. In general, our results showed that LV myocardial remodeling and contraction impairment in obese patients were responsible for RV subclinical dysfunction.

### Regional Fat Distributions and RV Dysfunction

Our study described the linear relationships between regional fat distributions and RV functional assessments, and the results showed that central fat distribution indicators (android fat% and trunk fat%) had deleterious effects on subclinical RV function, whereas peripheral fat distribution indicator (gynoid fat%) had a positive effect on it. Notably, our result showed that visceral fat and EAT volume were negatively associated with RV strain in univariate analysis, while no linear relationship with RV strain parameter in multivariate analysis. Previous CMR studies on obesity with no complications have reported that visceral fat, EAT volume or area had linear relationships with LV subclinical dysfunction in multivariate analysis (26, 27). However, whether EAT could predict RV subclinical dysfunction in these patients is still unclear. Additionally, different from the previous studies, we added DXA-related regional fat distributions (android fat, gynoid fat, trunk fat, peripheral fat) into multivariate analysis and they may attenuate the effects of EAT and visceral fat on the RV function. In other words, instead of EAT and visceral fat, trunk fat, android fat or gynoid fat were better predictors of RV subclinical function.

One explanation for the different effects of regional fat distributions on RV function was dyslipidemia. Our study revealed that central obesity parameters (android fat and trunk fat) were positively correlated with triglycerides, total cholesterol, and LDL, while were negatively correlated with HDL. The result is consistent with previous researches which demonstrated that central obesity parameters (android fat or trunk fat distributions) had positive associations with triglycerides and LDL levels, while they had negative associations with HDL levels (28–30). In contrast, we found that gynoid fat distribution had negative associations with triglycerides, total cholesterol, and LDL, while had a positive association with HDL. A previous study has also indicated that gynoid fat% had a negative association with hypertriglyceridemia (31, 32). It is well known that high HDL levels are considered as cardiovascular protective factors, while triglycerides, cholesterol, and LDL levels are considered cardiovascular risk factors. Obesity has also been shown to be associated with increased oxidized lipid levels (33), and oxidized LDL levels and oxidized lipid derivatives were shown to cause myocardial dysfunction and cardiomyopathy by inducing inflammation, apoptosis, and endoplasmic reticulum stress (34). Anti-oxidation proteins, such as platelet activating factor acetyl hydrolase and paraoxonase, are primarily included in HDL and might mitigate LDL oxidation and remove lipid oxidation products (35). Android fat and trunk fat have also been shown to be important risk factors for insulin resistance (30, 36). A previous study reported that insulin resistance was only found in the women with android obesity, rather than those with gynoid obesity (37). A study on sucrose-fed rats suggested that insulin resistance directly induced cardiac contractile impairments (38), and a clinical study in obese female subjects showed that its link with myocardial fatty acid metabolism could reduce myocardial contractile function (39). Adipokines and chronic inflammatory factors have also been implicated in obesity. Trunk fat and android fat were inversely correlated with adiponectin (40, 41), whereas correlated with increased leptin (42, 43). The leg fat was positively correlated with adiponectin (44). Adiponectin and leptin are proteins secreted by adipose tissue. Adiponectin is associated with antidiabetic and antiatherogenic properties, which mediates insulin-sensitizing effects and reduce hyperlipidemia (45, 46). It is well known that the leptin has beneficial effects on the inhibition of food intake and insulin sensitivity enhancements. However, high circulating leptin levels in obese individuals were mainly caused by leptin resistance, which was associated with increased insulin resistance (47, 48). The individuals with android obesity had elevated necrosis factor alpha-a (TNF-α) (49). The trunk fat% or android fat% was positively associated with TNF-α, while lower-body fat% or gynoid fat% was negatively associated with it (50). Leg fat was negatively associated with inflammatory markers including interleukin-6 (IL-6), C-reactive protein (CRP), and TNF-α (44, 51). CRP and TNF-α are associated with insulin resistance and atherosclerosis (45, 46, 52). IL-6 has been shown to have a critical role in aldosterone-induced macrophage recruitment and infiltration of myocardial macrophages, which play an important role in myocardial fibrosis (53).

In addition, android fat was correlated with higher blood pressure (29, 41), while the leg fat mass was negatively correlated with SBP (44). A study observing the relationship between blood pressure and RV function using STE demonstrated that subclinical RV function, assessed by strain, was impaired by increased blood pressure even though blood pressure was within the normal range (54).

### Limitations

There are several limitations in this study. First, this was a cross-sectional study, and we could not determine whether subtle RV contractile impairments cause heart failure with obesity progression and whether these changes can be reversed. Therefore, longitudinal studies are needed to examine dynamic cardiac changes and explore the underlying factors that reverse these changes. Second, past studies have indicated that multiple key inflammatory mediators and insulin resistance are consistently associated with obesity and obesity-related comorbidities. However, we did not examine inflammatory markers and insulin resistence in this study. These laboratory data should be measured and discussed in subsequent studies to clarify the mechanisms of myocardial damage in obese patients.

## Conclusions

Extensive subclinical RV dysfunction is found in uncomplicated obese adults. BMI, as an index of overall obesity, is independently associated with subclinical RV dysfunction. In addition, central obesity (android fat and trunk fat distributions) has a negative effect on subclinical RV function, while peripheral obesity (gynoid fat distribution) may have a positive effect on it.

## Data Availability Statement

The raw data supporting the conclusions of this article will be made available by the authors, without undue reservation.

## Ethics Statement

The studies involving human participants were reviewed and approved by the Institutional Review Board of the West China Hospital in Sichuan University. The patients/participants provided their written informed consent to participate in this study.

## Author Contributions

JLiu, JLi, LP, and NT: conception and design and provision of study materials or patients. LP and NT: administrative support and final approval of manuscript. JLiu, JLi, LP, HP, WH, XL, and XZ: collection and assembly of data. JLiu, JLi, and LP: manuscript writing. All authors: data analysis and interpretation.

## Funding

This work was supported by the National Natural Science Foundation of China (Grant Number 81601462), the Key Research & Development Project of Science and Technology of Sichuan Province (Grant Number 2021YFS0142), and 1.3.5. project for disciplines of excellence, West China Hospital, Sichuan University (Grant Number ZYGD18017).

## Conflict of Interest

XZ was employed by Siemens Healthineers Ltd. The remaining authors declare that the research was conducted in the absence of any commercial or financial relationships that could be construed as a potential conflict of interest.

## Publisher's Note

All claims expressed in this article are solely those of the authors and do not necessarily represent those of their affiliated organizations, or those of the publisher, the editors and the reviewers. Any product that may be evaluated in this article, or claim that may be made by its manufacturer, is not guaranteed or endorsed by the publisher.

## References

[1] Collaborators GBDO Afshin A Forouzanfar MH Reitsma MB Sur P Estep K. Health effects of overweight and obesity in 195 countries over 25 years. N Engl J Med. (2017) 377:13–27. 10.1056/NEJMoa1614362 28604169 PMC5477817

[2] Swinburn BA Kraak VI Allender S Atkins VJ Baker PI Bogard JR. The global syndemic of obesity, undernutrition, and climate change: the lancet commission report. Lancet. (2019) 393:791–846. 10.1016/S0140-6736(18)32822-8 30700377

[3] Tian Y Jiang C Wang M Cai R Zhang Y He Z. BMI, leisure-time physical activity, and physical fitness in adults in China: results from a series of national surveys, 2000-14. Lancet Diabetes Endocrinol. (2016) 4:487–97. 10.1016/S2213-8587(16)00081-4 27133172

[4] Unlu S Tacoy G. Early adulthood obesity is associated with impaired left ventricular and right ventricular functions evaluated by speckle tracking and 3D echocardiography. Turk Kardiyol Dern Ars. (2021) 49:312–20. 10.5543/tkda.2021.57336 34106065

[5] Orhan AL Uslu N Dayi SU Nurkalem Z Uzun F Erer HB. Effects of isolated obesity on left and right ventricular function: a tissue doppler and strain rate imaging study. Echocardiogr J Card. (2010) 27:236–43. 10.1111/j.1540-8175.2009.01024.x 20070359

[6] DeFaria Yeh D Stefanescu Schmidt AC Eisman AS Serfas JD Naqvi M Youniss MA. Impaired right ventricular reserve predicts adverse cardiac outcomes in adults with congenital right heart disease. Heart. (2018) 104:2044–50. 10.1136/heartjnl-2017-312572 30030334

[7] Wong CY O'Moore-Sullivan T Leano R Hukins C Jenkins C Marwick TH. Association of subclinical right ventricular dysfunction with obesity. J Am Coll Cardiol. (2006) 47:611–6. 10.1016/j.jacc.2005.11.015 16458145

[8] Nakanishi K Daimon M Yoshida Y Ishiwata J Sawada N Hirokawa M. Relation of body mass index to adverse right ventricular mechanics. Am J Cardiol. (2021) 144:137–42. 10.1016/j.amjcard.2020.12.069 33385349

[9] Pedrizzetti G Claus P Kilner PJ Nagel E. Principles of cardiovascular magnetic resonance feature tracking and echocardiographic speckle tracking for informed clinical use. J Cardiovasc Magn Reson. (2016) 18:51. 10.1186/s12968-016-0269-7 27561421 PMC5000424

[10] Grothues F Smith GC Moon JC Bellenger NG Collins P Klein HU. Comparison of interstudy reproducibility of cardiovascular magnetic resonance with two-dimensional echocardiography in normal subjects and in patients with heart failure or left ventricular hypertrophy. Am J Cardiol. (2002) 90:29–34. 10.1016/S0002-9149(02)02381-0 12088775

[11] Claus P Omar AMS Pedrizzetti G Sengupta PP Nagel E. Tissue tracking technology for assessing cardiac mechanics: principles, normal values, and clinical applications. JACC Cardiovasc Imaging. (2015) 8:1444–60. 10.1016/j.jcmg.2015.11.001 26699113

[12] Jing LY Pulenthiran A Nevius CD Mejia-Spiegeler A Suever JD Wehner GJ. Impaired right ventricular contractile function in childhood obesity and its association with right and left ventricular changes: a cine DENSE cardiac magnetic resonance study. J Cardiovasc Magn Reson. (2017) 19:49. 10.1186/s12968-017-0363-5 28659144 PMC5490166

[13] Sawada N Nakanishi K Daimon M Yoshida Y Ishiwata J Hirokawa M. Influence of visceral adiposity accumulation on adverse left and right ventricular mechanics in the community. Eur J Prev Cardiol. (2020) 27:2006–15. 10.1177/2047487319891286 31795766

[14] Gonzales JU Hadri O. Role of heart rate in the relation between regional body fat and subendocardial viability ratio in women. Clin Exp Pharmacol Physiol. (2016) 43:789–94. 10.1111/1440-1681.12597 27220028

[15] Chen YJ Peng Q Yang Y Zheng SS Wang Y Lu WL. The prevalence and increasing trends of overweight, general obesity, and abdominal obesity among Chinese adults: a repeated cross-sectional study. BMC Public Health. (2019) 19:1293. 10.1186/s12889-019-7633-0 31615464 PMC6794823

[16] Petak S Barbu CG Yu EW Fielding R Mulligan K Sabowitz B. The Official Positions of the International Society for Clinical Densitometry: body composition analysis reporting. J Clin Densitom. (2013) 16:508–19. 10.1016/j.jocd.2013.08.018 24183640

[17] Scatteia A Baritussio A Bucciarelli-Ducci C. Strain imaging using cardiac magnetic resonance. Heart Fail Rev. (2017) 22:465–76. 10.1007/s10741-017-9621-8 28620745 PMC5487809

[18] Chahal H McClelland RL Tandri H Jain A Turkbey EB Hundley WG. Obesity and right ventricular structure and function: the MESA-Right Ventricle Study. Chest. (2012) 141:388–95. 10.1378/chest.11-0172 21868467 PMC3277293

[19] Selthofer-Relatic K Bosnjak I Kibel A. Obesity related coronary microvascular dysfunction: from basic to clinical practice. Cardiol Res Pract. (2016) 2016:8173816. 10.1155/2016/8173816 27092288 PMC4820617

[20] Kovacs A Lakatos B Tokodi M Merkely B. Right ventricular mechanical pattern in health and disease: beyond longitudinal shortening. Heart Fail Rev. (2019) 24:511–20. 10.1007/s10741-019-09778-1 30852772 PMC6559995

[21] Chuang ML Danias PG Riley MF Hibberd MG Manning WJ Douglas PS. Effect of increased body mass index on accuracy of two-dimensional echocardiography for measurement of left ventricular volume, ejection fraction, and mass. Am J Cardiol. (2001) 87:371–4, A10. 10.1016/S0002-9149(00)01383-7 11165985

[22] Alpert MA Karthikeyan K Abdullah O Ghadban R. Obesity and cardiac remodeling in adults: mechanisms and clinical implications. Prog Cardiovasc Dis. (2018) 61:114–23. 10.1016/j.pcad.2018.07.012 29990533

[23] Alpert MA. Obesity cardiomyopathy: pathophysiology and evolution of the clinical syndrome. Am J Med Sci. (2001) 321:225–36. 10.1097/00000441-200104000-00003 11307864

[24] Damiano RJ Jr La Follette P Jr Cox JL Lowe JE Santamore WP. Significant left ventricular contribution to right ventricular systolic function. Am J Physiol. (1991) 261:H1514-24. 10.1152/ajpheart.1991.261.5.H1514 1951739

[25] Hoffman D Sisto D Frater RW Nikolic SD. Left-to-right ventricular interaction with a noncontracting right ventricle. J Thorac Cardiovasc Surg. (1994) 107:1496–502. 10.1016/S0022-5223(12)70150-2 8196395

[26] Lan Z Shengjia G Qingrou W Xiaoyue Z Simin W Caixia F. Left ventricular myocardial deformation: a study on diastolic function in the Chinese male population and its relationship with fat distribution. Quant Imaging Med Surg. (2020)10:634-45. 10.21037/qims.2020.01.16 32269924 PMC7136744

[27] Jing L Binkley CM Suever JD Umasankar N Haggerty CM Rich J. Cardiac remodeling and dysfunction in childhood obesity: a cardiovascular magnetic resonance study. J Cardiovasc Magn Reson. (2016) 18:28. 10.1186/s12968-016-0247-0 27165194 PMC4863365

[28] Latt E Maestu J Jurimae J. Longitudinal associations of android and gynoid fat mass on cardiovascular disease risk factors in normal weight and overweight boys during puberty. Am J Hum Biol. (2018) 30:e23171. 10.1002/ajhb.23171 30099806

[29] Daniels SR Morrison JA Sprecher DL Khoury P Kimball TR. Association of body fat distribution and cardiovascular risk factors in children and adolescents. Circulation. (1999) 99:541–5. 10.1161/01.CIR.99.4.541 9927401

[30] He F Rodriguez-Colon S Fernandez-Mendoza J Vgontzas AN Bixler EO Berg A. Abdominal obesity and metabolic syndrome burden in adolescents–Penn State Children Cohort study. J Clin Densitom. (2015) 18:30–6. 10.1016/j.jocd.2014.07.009 25220887 PMC4314452

[31] Wiklund P Toss F Weinehall L Hallmans G Franks PW Nordstrom A. Abdominal and gynoid fat mass are associated with cardiovascular risk factors in men and women. J Clin Endocrinol Metab. (2008) 93:4360–6. 10.1210/jc.2008-0804 18728169

[32] Wiklund P Toss F Jansson JH Eliasson M Hallmans G Nordstrom A. Abdominal and gynoid adipose distribution and incident myocardial infarction in women and men. Int J Obes. (2010) 34:1752–8. 10.1038/ijo.2010.102 20498655

[33] Marin MT Dasari PS Tryggestad JB Aston CE Teague AM Short KR. Oxidized HDL and LDL in adolescents with type 2 diabetes compared to normal weight and obese peers. J Diabetes Complications. (2015) 29:679–85. 10.1016/j.jdiacomp.2015.03.015 25881918 PMC9549762

[34] Hajri T. Effects of oxidized lipids and lipoproteins on cardiac function. Front Biosci. (2018) 23:1822–47. 10.2741/4675 29772531

[35] Kontush A de Faria EC Chantepie S Chapman MJ. Antioxidative activity of HDL particle subspecies is impaired in hyperalphalipoproteinemia: relevance of enzymatic and physicochemical properties. Arterioscler Thromb Vasc Biol. (2004) 24:526–33. 10.1161/01.ATV.0000118276.87061.00 14739123

[36] Aucouturier J Meyer M Thivel D Taillardat M Duche P. Effect of android to gynoid fat ratio on insulin resistance in obese youth. Arch Pediatr Adolesc Med. (2009) 163:826–31. 10.1001/archpediatrics.2009.148 19736336

[37] Orbetzova MM Koleva DI Mitkov MD Atanassova IB Nikolova JG Atanassova PK. Adipocytokines, neuropeptide Y and insulin resistance in overweight women with gynoid and android type of adipose tissue distribution. Folia Med. (2012) 54:22–9. 10.2478/v10153-011-0093-7 23270203

[38] Hintz KK Aberle NS Ren J. Insulin resistance induces hyperleptinemia, cardiac contractile dysfunction but not cardiac leptin resistance in ventricular myocytes. Int J Obes Relat Metab Disord. (2003) 27:1196–203. 10.1038/sj.ijo.0802389 14513067

[39] Peterson LR Herrero P Schechtman KB Racette SB Waggoner AD Kisrieva-Ware Z. Effect of obesity and insulin resistance on myocardial substrate metabolism and efficiency in young women. Circulation. (2004) 109:2191–6. 10.1161/01.CIR.0000127959.28627.F8 15123530

[40] Kouda K Nakamura H Ohara K Fujita Y Iki M. Increased ratio of trunk-to-appendicular fat and decreased adiponectin: a population-based study of school children in Hamamatsu, Japan. J Clin Densitom. (2017) 20:66–72. 10.1016/j.jocd.2015.10.004 26655234

[41] Miazgowski T Safranow K Krzyzanowska-Swiniarska B Iskierska K Widecka K. Adiponectin, visfatin and regional fat depots in normal weight obese premenopausal women. Eur J Clin Invest. (2013) 43:783–90. 10.1111/eci.12106 23650969

[42] Scott HA Gibson PG Garg ML Pretto JJ Morgan PJ Callister R. Relationship between body composition, inflammation and lung function in overweight and obese asthma. Respir Res. (2012)13:10. 10.1186/1465-9921-13-10 22296721 PMC3329414

[43] Cho BA Iyengar NM Zhou XK Mendieta H Winston L Falcone DJ. Increased trunk fat is associated with altered gene expression in breast tissue of normal weight women. NPJ Breast Cancer. (2022) 8:15. 10.1038/s41523-021-00369-8 35087024 PMC8795267

[44] Samouda H De Beaufort C Stranges S Hirsch M Van Nieuwenhuyse JP Dooms G. Cardiometabolic risk: leg fat is protective during childhood. Pediatr Diabetes. (2016) 17:300–8. 10.1111/pedi.12292 26083149

[45] Noordam R Boersma V Verkouter I le Cessie S Christen T Lamb HJ. The role of C-reactive protein, adiponectin and leptin in the association between abdominal adiposity and insulin resistance in middle-aged individuals. Nutra Metab Cardiovasc Dis. (2020) 30:1306–14. 10.1016/j.numecd.2020.04.021 32507340

[46] Lau WB Ohashi K Wang Y Ogawa H Murohara T Ma XL. Role of adipokines in cardiovascular disease. Circ J. (2017) 81:920–8. 10.1253/circj.CJ-17-0458 28603178

[47] Thorand B Zierer A Baumert J Meisinger C Herder C Koenig W. Associations between leptin and the leptin / adiponectin ratio and incident Type 2 diabetes in middle-aged men and women: results from the MONICA / KORA Augsburg study 1984-2002. Diabet Med. (2010) 27:1004–11. 10.1111/j.1464-5491.2010.03043.x 20722673

[48] Sainz N Barrenetxe J Moreno-Aliaga MJ Martinez JA. Leptin resistance and diet-induced obesity: central and peripheral actions of leptin. Metabolism. (2015) 64:35–46. 10.1016/j.metabol.2014.10.015 25497342

[49] Bal Y Adas M Helvaci A. Evaluation of the relationship between insulin resistance and plasma tumor necrosis factor-alpha, interleukin-6 and C-reactive protein levels in obese women. Bratisl Lek Listy. (2010) 111:200–4. 10.3357/ASEM.21011.2010 20586146

[50] Wu B Huang J Fukuo K Suzuki K Yoshino G Kazumi T. Different associations of trunk and lower-body fat mass distribution with cardiometabolic risk factors between healthy middle-aged men and women. Int J Endocrinol. (2018) 2018:1289485. 10.1155/2018/1289485 29531527 PMC5817354

[51] Koster A Stenholm S Alley DE Kim LJ Simonsick EM Kanaya AM. Body fat distribution and inflammation among obese older adults with and without metabolic syndrome. Obesity. (2010) 18:2354–61. 10.1038/oby.2010.86 20395951 PMC3095947

[52] Borst SE. The role of TNF-alpha in insulin resistance. Endocrine. (2004) 23:177–82. 10.1385/ENDO:23:2-3:177 15146098

[53] Liao CW Chou CH Wu XM Chen ZW Chen YH Chang YY. Interleukin-6 plays a critical role in aldosterone-induced macrophage recruitment and infiltration in the myocardium. Biochim Biophys Acta Mol Basis Dis. (2020) 1866:165627. 10.1016/j.bbadis.2019.165627 31785407

[54] Pedrinelli R Canale ML Giannini C Talini E Dell'Omo G Di Bello V. Abnormal right ventricular mechanics in early systemic hypertension: a two-dimensional strain imaging study. Eur J Echocardiogr. (2010) 11:738–42. 10.1093/ejechocard/jeq059 20472915

